# Supplementary material for: A systematic search for RNA structural switches across the human transcriptome
Source: Nat Methods. 2024 Jul 16;21(9):1634–45. doi: 10.1038/s41592-024-02335-1 (PMC11399106; doi:10.1038/s41592-024-02335-1)
Supplement: Supplementary file 1 — Supplementary Protocol [file 41592_2024_2335_MOESM1_ESM.pdf]

nature methods

Supplementary information https://doi.org/10.1038/s41592-024-02335-1

A systematic search for RNA structural switches across the human transcriptome

In the format provided by the authors and unedited

The PDF file includes:

Supplementary protocol 1.

Other Supplementary Material for this manuscript includes the following:

Extended Data Figure 1: SwitchFinder identifies saddles in RNA folding energy landscape
Extended Data Figure 2: Overview of high-throughput screening approaches for improved RNA switch predictions
Extended Data Figure 3: In vitro SHAPE reactivity of the RORC RNA switch sequence in vitro.
Extended Data Figure 4: Qualitative modeling of cryoEM data
Extended Data Figure 5: CryoEM image processing and validation
Extended Data Figure 6: Differentiation of Th17 cells from primary human CD4+ cells
Extended Data Figure 7: CRISPRi screen highlights the pathways acting downstream of the RORC RNA switch

Data file S1 (Microsoft Excel format). AUC values for SwitchFinder prediction of RNA switches from common riboswitch Rfam families
Data file S2 (Microsoft Excel format). SwitchFinder predictions for the 3,750 RNA fragments selected for further in vivo screening
Data file S3 (Microsoft Excel format). mRNA and gDNA read counts in the sorted bins of the functional screen
Data file S4 (Microsoft Excel format). DMS-MaPseq reactivity profiles and the second iteration of SwitchFinder predictions for 1,454 high confidence RNA switches
Data file S5 (Microsoft Excel format). mRNA and gDNA read counts in the sorted bins for the massively parallel mutagenesis analysis
Data file S6 (Microsoft Excel format). Sequences of RORC mutant sequences referred to in the paper
Data file S7 (Microsoft Excel format). Cryo-EM data collection, refinement and validation statistics
Data file S8 (Microsoft Excel format). Cryo-EM data collection parameters
Data file S9 (Microsoft Excel format). Antisense oligonucleotides (ASO) used for RORC structural ensemble perturbation
Data file S10 (Microsoft Excel format). gDNA read counts for the CRISPRi screens

1. Prediction of candidate RNA switches

Prerequisites:

- Working installation of Docker. Follow the [official installation guide](#).
- Up to date installation of pip. Pip can be upgraded with the command `pip install --upgrade pip setuptools wheel`
- A set of sequences of interest in fasta format

Steps:

- Pull a docker container image with the following command (Running the command might require “sudo” depending on your user privileges)

```
docker pull goodarzilaborder/switch_finder_image:latest
```

- Launch the Docker image

```
docker run -it -v $(pwd):/switchfinder
goodarzilaborder/switch_finder_image:latest
```

- Install the Python code inside the Docker container

```
pip install git+https://github.com/goodarzilab/SwitchFinder.git
```

- Download example data (within the Docker container):

```
wget
https://raw.githubusercontent.com/goodarzilab/SwitchFinder/main/example_dat
a/example_sequences.fa
```

- Run the pipeline

```
SwitchFinder_pipeline \
        --input_fastafile example_sequences.fa \
        --out output \
        --temp_folder temp \
        --RNAstructure_path $RNAstructure_path \
        --RNApathfinder_path $RNApathfinder_path
```

- Move the output files to the desired location (outside of Docker container)

2. Library ordering and cloning

The protocol generally follows the library cloning protocol by [4]

- Select the sequences that you would like to test based on SwitchFinder output: find the sequences in the *RNA_switch_structures.txt* file and the scores in the *RNA_switch_scores.txt* file

- Include the mutations that shift the equilibrium of the two conformation into the library. The sequences are listed in the *generated_mutations.txt* file. In this case, each candidate RNA switch is represented by 5 sequences:
  - Reference sequence
  - Major Strengthen: Enhances the stability of the first conformation.
  - Second Strengthen: Enhances the stability of the second conformation.
  - Second Weaken: Reduces the stability of the second conformation.
  - Major Weaken: Reduces the stability of the first conformation.

- Prepare the library sequences by adding the restriction sites and PCR adapters to both ends of the sequences:
  - For testing 3'UTRs: TCCCTTGGAGAACCACCTTG**ACGCGT**-INSERT-**TTAATTAA**TAAGCTGGAAACAGCATAGCAAG
  - For testing 5'UTRs: TCCCTTGGAGAACCACCTTG**GGTCTC**n**CGTC**-AGATC-INSERT-gccacc-**ATGG**n**GAGACC**TAAGCTGGAAACAGCATAGCAAG
  - In orange are suggested PCR adapters; in blue are suggested restriction sites; in green is the Kozak sequence; in red is the CMV initiator). One can also use any of the PCR adapters recommended by [4]
  - Order the oligonucleotide library synthesis from Twist Biosciences, Agilent, IDT, or any other provider
- Amplify the libraries following manufacturer's recommendations, e.g. Amplifying Twist Oligo Pools. We strongly recommend using the minimal number of cycles possible in order to avoid amplification bias.
- Digest the cloning vector:
  - For testing 3'UTR: use pMK053 (Addgene #213963). Digest with the mix of enzymes rSAP (NEB), MluI (NEB), PacI (NEB), following manufacturer's recommendations. Test the digestion by running the digested and the undigested enzyme on a 0.8% agarose gel.
  - For testing 5'UTR: use pMK089 (Addgene #213964). Digest with the mix of enzymes rSAP (NEB) and BspMI (NEB), following manufacturer's recommendations. Test the digestion by running the digested and the undigested enzyme on a 0.8% agarose gel.
  - After the digestion, purify the DNA with the DNA Clean & Concentrator-5 kit (Zymo).
- Digest the amplified oligonucleotide library:
  - For testing 3'UTR: if using the amplicon design listed above, digest with the mix of enzymes MluI (NEB) and PacI (NEB), following manufacturer's recommendations.
  - For testing 5'UTR: if using the amplicon design listed above, digest with BsaI (NEB), following manufacturer's recommendations.
- Purify the digested oligonucleotide library:
  - Run the digested library on a polyacrylamide gel, cut out the band, and extract the DNA from the band as described in [4]. Depending on the size of the insert, adjust the choice of the gel. For example: for 240 nt long inserts use 6% gel, for

the 50 nt inserts use 10% gel. Pre-made Novex TBE Gels can be purchased from ThermoFisher.

- Ligate the insert and the vector:
  - Set up a ligation using 1:1 vector:insert molar ratio, using T4 DNA ligase (NEB), following manufacturer's recommendations. Additionally, set up a 1:0 vector:insert reaction as a self-ligation control. Using 40 fmol vector and 40 fmol insert per reaction is a good starting point.
- Purify the ligation product
  - Perform ethanol precipitation as described in [4]. Important: resuspend in 2 ul TE buffer only.
- Transform the ligation product into competent *E. coli*.
  - If the oligonucleotide library contains less than 1.000 candidate sequences, it is possible to use chemical transformation. Otherwise, do electroporation, using either MegaX competent cells (Thermo Fisher), or Endura Competent Cells (LGC), following the manufacturer's recommendations.
  - After the 1 hour outgrowth step, take out 0.05% of the outgrowth culture, prepare 100X, 1000X, and 10.000X dilutions, and seed 50 ul from each dilution onto an LB Agar Ampicillin plate. Additionally, electroporate and seed the negative control. Add the rest of the outgrowth culture to a maxi-prep LB culture (75 to 150 mL volume).
  - Next day, estimate the number of colonies on the 100X, 1000X, and 10.000X dilutions plates, and use it to approximate the number of colony-forming units (CFUs) in the original outgrowth culture. Ensure the number is at least 100X higher than the number of sequences in the ordered oligonucleotide library. Additionally, perform colony Sanger sequencing of 10 colonies from the 100X, 1000X, or 10.000X plates. Ensure all the 10 colonies map to different sequences from the original oligonucleotide library. For pMK053 insert Sanger sequencing, we use primers CTGCTAGCTAGATGACTAAACGC and GTGGTCTGGATCCACCGGTCC. For pMK089 insert Sanger sequencing, we use primers GAGCTCGTTTAGTGAACCGTCAG and CCGGTGGTGCAGATGAACTTC
- Prepare the plasmid library using ZymoPURE II Plasmid Maxiprep (Zymo Research).

3. Massively Parallel Reporter Assay

- Prepare the lentivirus using the plasmid library as described in [5]. Aliquot the virus and freeze the aliquots at -80C. Each aliquot is only good for one use. Scale up depending on the library size and the number of assays planned.
- Infect the cell line of interest with lentivirus.
  - Follow the Weissmann lab protocols for suspension or adherent cells. The individual steps involve titering the virus, infecting the cells, measuring the infection rate, performing the puromycin selection and recovery. Important: ensure the infection rate on the days 2-3 after the infection is less than 30%. When using pMK053 (Addgene #213963), the infection rate is estimated by

measuring the fraction of mCherry-positive cells. When using pMK089 (Addgene #213964), the infection rate is estimated by measuring the fraction of eGFP-positive cells. The timeline needs to be adjusted depending on the specific cell type of interest and can take between 6-12 days. The cells are ready for the next step once they are fully recovered after the puromycin selection (>90% viability) and the fraction of cells expressing the fluorescent marker is >90%.

- Perform FACS sorting. This protocol is written for the BD instruments.
    - Setting Up Sorting Scheme
        - Create a new sorting experiment using a basic layout template.
        - Configure the default settings, so that the cells represent a visible cloud on the FCS-SSC plot, the doublets are easily distinguishable on the FSC-A-FSC-W plot, and the cell population forms a diagonal cloud on the FSC-A-FSC-H plot.
        - Add the eGFP/mCherry ratio to the Global Worksheet in the Cytometer window.
    - Calibration Using Control Samples
        - Calibrate the laser voltages using a negative control sample. Load the control sample and adjust the lasers as needed so that both eGFP and mCherry fluorescence histograms are centered above the 10^2 value
    - Calibration Using Infected Sample
        - Set the gates for the live cells (FCS-SSC), single cells (FSC-A-FSC-W), and eGFP+ cells (in case of pMK089) or mCherry+ cells (in case of pMK053).
        - Calibrate the GFP/mCherry scaling using an infected sample. Adjust the scaling for the eGFP/mCherry ratio so that a minimal number of cells falls outside the eGFP/mCherry histogram.
        - Establish four quantile gates for sorting based on the GFP/mCherry ratio histogram.
        - In the sorting window, set up the sort layout for precision purity (e.g., 4-way purity) and choose the appropriate device (e.g., 4-way device).
        - Check for proper alignment of sorting streams. Adjust the flows to ensure they target the centers of the tubes. Avoid confusion between inner and outer values.
    - Sample Preparation and Sorting
        - Add 1 mL of FACS buffer (1% FBS, 1 mM EDTA in PBS) to the output tubes and load them into the tube holder.
        - Initiate the sorting process. Adjust the Flow Rate to maintain an event rate just under 10k events per second.
        - Ensure that the sorting efficiency stays around 80%.
        - Conduct periodic checks for any clogging and ensure there is sufficient liquid in the input tube.
    - Handling Samples and Timeline

        - For each replicate, aim to sort the number of cells equal to 250X the number of sequences in the oligonucleotide library into each bin, but no less than 5 million cells per bin.
        - Manage the sorting of multiple replicates efficiently, switching input and output tubes as required.
    - Post-Sorting Procedures
        - After sorting, conduct a thorough clean-up of the sorter.
        - Pellet the sorted cells and transfer them to appropriate containers for further processing or storage.
- Library preparation
    - Design and order the appropriate primers
        - For pMK053, we use the following template:
            - AATGATACGGCGACCACCGAGATCTACACNNNNNNNNACACTCTTTCCCTACACGACGCTCTTCCGATCTXXXGTGGTCTGGATCCACCGGTCC, where brown is Illumina P5 adapter, purple is the i5 index, green is the TruSeq Read 1 primer site, orange is staggering region, dark blue is the primer binding downstream of the insert.
            - CAAGCAGAAGACGGCATACGAGATNNNNNNNNGTGACTGGAGTTCAGACGTGTGCTCTTCCGATCXXXACTGCTAGCTAGATGACTAAACGCGT, where brown is Illumina P7 adapter, purple is the i7 index, green is the TruSeq Read 2 primer site, orange is staggering region, dark blue is the primer binding upstream of the insert.
        - For pMK089, we use the following template:
            - AATGATACGGCGACCACCGAGATCTACACNNNNNNNNACACTCTTTCCCTACACGACGCTCTTCCGATCTXXXGAGCTCGTTTAGTGAACCGTCAGATC, where brown is Illumina P5 adapter, purple is the i5 index, green is the TruSeq Read 1 primer site, orange is staggering region, dark blue is the primer binding downstream of the insert.
            - CAAGCAGAAGACGGCATACGAGATNNNNNNNNGTGACTGGAGTTCAGACGTGTGCTCTTCCGATCXXXCCGGTGGTGCAGATGAACTTC, where brown is Illumina P7 adapter, purple is the i7 index, green is the TruSeq Read 2 primer site, orange is staggering region, dark blue is the primer binding upstream of the insert.
        - We recommend designing the primers in such way that each sample is amplified with a unique combination of i5 and i7 indexes. The example index sequences can be found in the Illumina Adapter Sequences.
        - We also recommend ordering multiple primers with varying number of bases within the staggering region, as described in [6], to ensure the cluster diversity on the Illumina sequences.

- Extract genomic DNA from the pelleted cells using NucleoSpin Blood kit from Macherey Nagel, according to manufacturer's protocol. Choose the approximate size of the kit according to the number of cells sorted. For example, for the samples between 5 to 20 million cells, use NucleoSpin Blood L. If many samples are processed at one, use NucleoSpin Blood L Vacuum kit.
- Determine the necessary number of cycles with RT-qPCR
  - qPCR Mixture Composition: For each 20 ul reaction, mix 10 µl of NEBNext Ultra II Q5 Master Mix, 1 µl of each primer (Forward and Reverse, 10 uM), 0.5 µl of 25 µM ROX (Thermo Fisher), 0.5 µl of 50X SYBR-Gold (diluted from SYBR™ Gold Nucleic Acid Gel Stain, 10,000X Concentrate in DMSO, Thermo Fisher), and gDNA template up to 20 µl total volume with nuclease-free water.
  - Distribute the mixture into a qPCR plate and thermocycle in the appropriate RT-qPCR machine for 30 cycles.
    - Step # Temperature Time
    - 1 98 °C 30 s
    - 2 98 °C 10 s
    - 3 69 °C 20 s
    - 4 72 °C 20 s
    - 5 come back to step 2: 30 times
    - 6 72 °C 5 m
  - Analyze amplification plots to define the optimal PCR cycle number, aiming for the midpoint of the exponential phase. In our experience, 24 cycles produce sufficient amount of product.
  - Analyze the amplified product on a 2% agarose gel with GeneRuler LR ladder (ThermoFisher). Ensure that the observed band corresponds to the expected size.
- PCR-amplify the inserts
  - Prepare the PCR mix for each sample as described above. For each 50 ul reaction, mix 25 µl of NEBNext Ultra II Q5 Master Mix, 2.5 µl of each primer (Forward and Reverse, 10 uM), gDNA template (2.5X the volume used in the test qPCR reaction), and up to 50 µl total volume with nuclease-free water. All the gDNA extracted previously needs to be amplified. Distribute into PCR plates, seal, and run amplification.
  - Amplify as above, using the determined number of cycles.
  - Post-amplification, pool the PCR products for each sample in labeled tubes. Store at -20°C.
- PCR product purification
  - Purify the product using the Select-a-Size DNA Clean & Concentrator MagBead Kit (Zymo Research). We recommend starting with 100 ul PCR product, doing two-sided purification, and adjusting the thresholds depending on the expected amplicon size. For example, for the amplicons of size 400 we recommend choosing the thresholds of 250 nt and 1000 nt, respectively.

- Quality control
  - We highly recommend using Agilent TapeStation or Agilent BioAnalyzer to ensure the purity and the right size of the amplified DNA.
- Pooling
  - Determine the DNA concentration in each sample with Qubit dsDNA Quantitation, High Sensitivity kit (Thermo Fisher)
  - Pool the samples at the equimolar ratio. Ensure each sample has a unique i5-i7 index combination.
- Sequence
  - Sequence the pooled sample on an appropriate sequencing machine. We recommend sequencing at a depth of >20 million reads per individual sample, and using 15% PhiX.

- Analysis
  - Remove the adaptor sequences.
    - Example command: `cutadapt -j 1 -g TGGTCTGGATCCACCGGTCCGGTTTAATTAA -G CTGCTAGCTAGATGACTAAACGCGTATGG -m 15 -o ${read_1_out_name} -p ${read_2_out_name} ${read_1_in_name} ${read_2_in_name} > ${log_file_name}`
    - Adjust the "-g" and "-G" arguments in the command according to the primer regions binding to the vector (marked blue in the examples given above). The current example is given for pMK053 vector-based primers.
  - Prepare the reference sequence database.
    - Open the FASTA file with the sequences of oligonucleotides that was sent to the DNA synthesis company
    - Remove the constant adapter regions surrounding the variable inserts.
    - Create bwa index file. Example command: `bwa index -p ${library_name} ${library_fasta_file}`
  - Map the reads
    - Example command: `bwa mem -t 1 $fasta_reference $read_1_name $read_2_name 2> ${log_file_name}`
    - Make sure at least 80% reads align exactly 1 time
  - Sort the reads
    - Example command: `samtools sort -o ${sorted_filename} ${input_filename} ; samtools index ${sorted_filename}`
  - Create a GTF annotation based on FASTA file with the sequences of oligonucleotides that was sent to a DNA synthesis company.
    - Example: read the fasta file into a dictionary, where the keys are the fragment names and the values are the sequences. Then write a GTF file using the following function:

```
def write_gft_contigs_file(tr_dict_loc, outfile):
    with open(outfile, 'w') as wf:
        for anno in sorted(list(tr_dict_loc.keys())):
```

```
            seqname = anno
            source = 'lib'
            feature = 'gene'
            start = str(1)
            end = str(len(tr_dict_loc[anno]))
            score = str(1)
            strand = '+'
            frame = str(0)
            attribute = 'gene_id "%s"; transcript_id "%s";' % (anno, anno)
            current_string = "%s\t%s\t%s\t%s\t%s\t%s\t%s\t%s\t%s\n" % (seqname,
source, feature,
                                                                      start, end,
score, strand,
                                                                      frame,
attribute)
            wf.write(current_string)


fragments_gtf_filename = "example.gtf"
write_gft_contigs_file(fragments_seq_dict, fragments_gtf_filename)
```

- Count the reads
    - Example command: `featureCounts -T 1 -t gene -a ${annotation_file} -o ${output_file}_counts.txt $i > $log_file_name`
    - Ensure the reads counts correlate well (>0.8) between replicates
- Normalize the read counts as in [7]

```
def geom_mean_1d(inp_array):
    x = np.log(inp_array[inp_array > 0]).sum() / inp_array.shape[0]
    return np.exp(x)
def normalize_median_of_ratios(inp_df, do_print = False):
    geom_means = inp_df.apply(geom_mean_1d, axis=1)
    ratios_df = inp_df.divide(geom_means, axis=0)
    norm_factors = ratios_df.median(axis=0)
    if do_print:
        print('Normalization factors: ')
        print(norm_factors)
    normalized_df = inp_df.divide(norm_factors, axis=1)
    return normalized_df
```

- Calculate mass centers across the read counts in the sorting bins
- Record measurements corresponding to normalized read counts in sorting bins for each candidate switch

- Candidate RNA Switch Ranking

- This step is only applicable if the mutations favoring conformation 1 or conformation 2 of the candidate RNA switch sequences were included into the oligonucleotide library.
- Correlation analysis:
  - Calculate correlations between the effects of mutations designed to favor similar or opposing conformations:
    - Correlation_Same_1: Correlation between Major and Second Strengthen mutations.
    - Correlation_Same_2: Correlation between Major and Second Weaken mutations.
    - Correlation_Opposite_1: Correlation between Major Strengthen and Second Weaken mutations.
    - Correlation_Opposite_2: Correlation between Major Strengthen and Second Strengthen mutations.
    - The correlations are calculated between the arrays of normalized read counts across the sorting bins.
- Switch scoring:
  - Score each candidate switch as: `Score = Mean(Correlation_Same_1, Correlation_Same_2) - Mean(Correlation_Opposite_1, Correlation_Opposite_2).`
  - Rank candidate RNA switches based on this score.
  - Identify significant switches based on scores exceeding a defined threshold (e.g., mean score + one standard deviation).

4. DMS-MaPseq

The protocol generally follows the DMS-MaPseq protocol by [8]

- Cautions before working with DMS:
  - DMS is highly toxic and should only be used in a well-ventilated fume hood. We recommend using multiple layers of nitrile gloves such that the exterior layer can be removed and replaced after handling DMS directly. DMS should have a faint yellow color, that will become darker due to oxidation over time. Open a fresh bottle of DMS every six months to ensure freshness and optimal reactivity. DMS will be quenched during the following procedure by BME and resulting liquid waste should be disposed of per your local hazardous waste regulations. With the exception of the centrifugation steps and final steps of total RNA extraction, all steps should be done in a fume hood.
- Perform DMS treatment as described in [8]:
  - Begin with a 15 cm2 plate of ~80% confluent MDA-LM2 cells in 15 ml media. Cells were harvested ~48hrs post-seeding.
  - Working a fume hood, remove 10 ml media from the plate into a 50 ml conical and add 250 μl DMS. Shake vigorously to ensure mixing.

- Slowly pipette the DMS/media mixture back on the plate by tilting the plate and pipetting into a corner.
- Plates incubated at room temp in fume hood for 7 min with occasional swirling.
- Carefully pipet out the DMS/media into designated waste.
- Add 10 ml of 30% v/v BME (diluted in 1 x PBS) and collect the cells into a 15 ml conical using a scraper to scrape the cells from the plate. Parafilm tubes before moving from hood.
- Centrifuge cells at 1000 x g at 4°C for 3 min; decant the BME solution into designated waste.
- Wash the cells by adding 10 ml of 1 x PBS and repeating the centrifugation step. Decant the PBS.
- Add 1 ml Trizol + 10ul BME to cell pellet, and pipet to lyse. Note: perform all steps at room temp unless otherwise indicated. Also collect total RNA from non-DMS treated cells by washing plates once with PBS, then lysing with 1mL Trizol in dish and proceeding with the below instructions. Note, that DMS-treated RNA can be brittle and high temperature or vortexing steps should be minimized to prevent fragmentation.
- Incubate for 5 min to permit complete dissociation of the nucleoproteins complex.
- Add 0.2mL of chloroform per 1mL Trizol used for lysis, vigorously shake tubes by hand for 15 sec, and then incubate 2-3 minutes.
- Centrifuge tubes at 12,000 x g at 4° C for 15 min. After spin, mixture should separate into a lower red phenol phase, an interphase, and a colorless upper aqueous phase. RNA remains exclusively in the aqueous phase. The volume of the aqueous phase is ~60% of the volume of Trizol used for lysis.
- Transfer aqueous phase to clean tube and precipitate RNA by adding isopropanol and mixing by inversion. Use 0.5mL of isopropanol per 1mL Trizol used for lysis. Incubate samples for 10 min and spin at 12,000 x g at 4°C for 10 min.
- Remove sup and wash RNA pellet once with 1mL 75% ethanol. Use at least 1mL 75% ethanol per 1mL Trizol used for lysis. Mix the sample by inversion and spin at 7,500 x g at 4°C for 5min.
- Air dry pellet then resuspend in 75ul H2O.
- Store RNA at -80.

- Perform DNase Treatment
  - Mix the following components in a clean microfuge tube: 20 ug total RNA (from previous step), 8 ul 10X TURBO DNase buffer, 4 ul TURBO DNase, H2O to 80ul total. Incubate 30 min at 37C.
  - Clean up with RNA Clean & Concentrator-25
  - Elute sample from the column with 25 µl RNase-free water. Add another 25 µl of water to the column and elute again into the same tube, collecting ~48 ul of final volume.
- Reverse Transcription
  - Perform reverse transcription using Induro Reverse Transcriptase (NEB), according to manufacturer’s recommendations. Use all the RNA from the

previous step. Use a gene-specific primer instead of the Random Primer Mix. For pMK053, we use the primer CTCTTTCCCTACACGACGCTCTTCCGATCTNNNNNNNNNNNtggtctggatccaccg gtccgg, where green is a part of the TruSeq primer site, grey is unique molecular identifier (UMI), dark blue is the primer binding downstream of the insert.
    - Purify the cDNA with the DNA Clean & Concentrator-25 kit (Zymo).
- Design and order the appropriate PCR primers
    - For pMK053, we use the following template:
        - AATGATACGGCGACCACCGAGATCTACACNNNNNNNNACACTCTTTC CCTACACGACGCTC, where brown is Illumina P5 adapter, purple is the i5 index, green is the TruSeq Read 1 primer site.
        - CAAGCAGAAGACGGCATACGAGATNNNNNNNNGTGACTGGAGTTCA GACGTGTGCTCTTCCGATCXXXACTGCTAGCTAGATGACTAAACGCG T, where brown is Illumina P7 adapter, purple is the i7 index, green is the TruSeq Read 2 primer site, orange is staggering region, dark blue is the primer binding upstream of the insert.
        - We recommend designing the primers in such way that each sample is amplified with a unique combination of i5 and i7 indexes. The example index sequences can be found in the Illumina Adapter Sequences.
        - We also recommend ordering multiple primers with varying number of bases within the staggering region, as described in [6], to ensure the cluster diversity on the Illumina sequences.This applies to the upstream primer only, since the downstream primer contains a variable UMI region.
- PCR-amplify
    - For each 50 ul reaction, mix 25 µl of NEBNext Ultra II Q5 Master Mix, 2.5 µl of each primer (Forward and Reverse, 10 uM), cDNA template (up to 5 ug total), and up to 50 µl total volume with nuclease-free water. All the cDNA from the previous step previously needs to be amplified. Distribute into PCR plates, seal, and run amplification for 30 cycles, following the manufacturer's recommendations. For the example primers listed above, we use the annealing temperature of 67C.
- Quality control and pooling - as described above
- Sequence the pooled sample on an appropriate sequencing machine.
- Analysis
    - Extract the UMIs.
        - Example command: `umi_tools extract --extract-method string --bc-pattern=NNNNNNNNNNN -I ${read_1_in_name} -S ${read_1_out_name} --read2-in=${read_2_in_name} --read2-out=${read_2_out_name} --log=${log_file_name}`
    - Remove the adaptor sequences.
        - Example command: `cutadapt -j 1 -g GTGGTCTGGATCCACCGGTCCggtttaattaa -G TGCTAGCTAGATGACTAAACGCGTATGG -m 15 -o ${read_1_out_name} -p`

`${read_2_out_name} ${read_1_in_name} ${read_2_in_name} > ${log_file_name}`

- Adjust the "-g" and "-G" arguments in the command according to the primer regions binding to the vector (marked blue in the examples given above). The current example is given for pMK053 vector-based primers.

- Merge the paired-end reads.
  - Example command: `pear --threads 1 --forward-fastq $read_2_name --reverse-fastq $read_1_name --output ${output_file}_pear_combined.bam > ${log_file_name}`
- Prepare the reference sequence database.
  - Open the FASTA file with the sequences of oligonucleotides that was sent to the DNA synthesis company
  - Remove the constant adapter regions surrounding the variable inserts.
  - Create bwa index file. Example command: `bwa index -p ${library_name} %{library_fasta_file}`
- Map the reads
  - Example command: `bwa mem -t 1 $fasta_reference $combined_read_name 2> ${log_file_name}`
  - Make sure at least 80% reads align exactly 1 time
- Sort the reads
  - Example command: `samtools sort -o ${sorted_filename} ${input_filename} ; samtools index ${sorted_filename}`
- Deduplicate the reads
  - Example command: `umi_tools dedup --buffer-whole-contig -I ${i} --output-stats=${dedup_log_name} -S ${dedup_output_file}`
- Sort the deduplicated reads
  - Example command: `samtools sort -@ 30 -o ${sorted_filename} ${dedup_output_file} ; samtools index ${sorted_filename}`
- Calculate and normalize single-nucleotide accessibility values
  - Use the functions provided in the dms_utils github.
  - Read the FASTA file (the file with the sequences of oligonucleotides that was sent to a DNA synthesis company) into a dictionary, where the keys are the fragment names and the values are the sequences ({fragments_seq_dict})
  - Encode the individual read alignments as bit assays, as in [9]. Example command: `bit_dict = dms_utils.reads_to_bitvector_arrays({sample_filename}, adapter_5_size=0, adapter_3_size=0, base_quality_threshold = 30)`
  - Calculate the mutation rates. Mask out the mutation rates for T and G nucleotides. Example command:

```
# Initialize dictionary to store mutation fractions for a specific sample
```

```
mutation_rates_dict = {}

for el in bit_dict:
    # Calculate mutation fractions
    current_frequencies =
dms_utils.caluclate_mutation_fractions(bit_dict[el])
    # Zero out non-A and non-C positions
    masks_AC_dict = dms_utils.mask_out_non_A_C_positions(bit_dict,
fragments_seq_dict)
    current_frequencies[np.invert(masks_AC_dict[el])] = 0
    # Update the dictionary
    mutation_rates_dict[el] = current_frequencies
```

- Normalize the mutation rates as in [10], setting the outlier threshold as 0.1 as in [11]. Set the reactivities for T and G nucleotides to negative values. Example command:

```
normalized_mutation_rates_dict =
dms_utils.apply_normalization_to_every_element(mutation_rates_dict)
normalized_mutation_rates_dict =
dms_utils.set_non_AC_reactivities_to_neg_values(normalized_mutation_rates_d
ict, masks_AC_dict)
```

- The single-nucleotide accessibility values can be formatted for informing the RNA folding software, such as RNAstructure or ViennaRNA, or to inform SwitchFinder's prediction of RNA switches.

1. Barsacchi, M., Novoa, E. M., Kellis, M. & Bechini, A. SwiSpot: modeling riboswitches by spotting out switching sequences. *Bioinformatics* **32**, 3252–3259 (2016).
2. Goodarzi, H., Elemento, O. & Tavazoie, S. Revealing global regulatory perturbations across human cancers. *Mol. Cell* **36**, 900–911 (2009).
3. Navickas, A. *et al.* An mRNA processing pathway suppresses metastasis by governing translational control from the nucleus. *Nat. Cell Biol.* **25**, 892–903 (2023).
4. Heo, S.-J. *et al.* Optimized CRISPR guide RNA library cloning reduces skew and enables more compact genetic screens. *bioRxiv* 2022.12.22.521524 (2022) doi:10.1101/2022.12.22.521524.

5. Saha, I. Lentivirus production v1. (2022) doi:10.17504/protocols.io.6qpvr4xn3gmk/v1.

6. Wohlhieter, C. A. *et al.* An optimized NGS sample preparation protocol for in vitro CRISPR screens. *STAR Protocols* **2**, 100390 (2021).

7. Anders, S. & Huber, W. Differential expression analysis for sequence count data. *Nature Precedings* 1–1 (2010).

8. Zubradt, M. *et al.* DMS-MaPseq for genome-wide or targeted RNA structure probing in vivo. *Nat. Methods* **14**, 75–82 (2017).

9. Tomezsko, P. J. *et al.* Determination of RNA structural diversity and its role in HIV-1 RNA splicing. *Nature* **582**, 438–442 (2020).

10. Low, J. T. & Weeks, K. M. SHAPE-directed RNA secondary structure prediction. *Methods* **52**, 150–158 (2010).

11. Hajdin, C. E. *et al.* Accurate SHAPE-directed RNA secondary structure modeling, including pseudoknots. *Proc. Natl. Acad. Sci. U. S. A.* **110**, 5498–5503 (2013).
