# Supplementary material for: A systematic search for RNA structural switches across the human transcriptome
Source: Nat Methods. 2024 Jul 16;21(9):1634–45. doi: 10.1038/s41592-024-02335-1 (PMC11399106; doi:10.1038/s41592-024-02335-1)
Supplement: Supplementary file 2 — Reporting Summary [file 41592_2024_2335_MOESM2_ESM.pdf]

nature portfolio

Corresponding author(s): Hani Goodarzi

Last updated by author(s): 04-30-2024

Reporting Summary

Nature Portfolio wishes to improve the reproducibility of the work that we publish. This form provides structure for consistency and transparency in reporting. For further information on Nature Portfolio policies, see our Editorial Policies and the Editorial Policy Checklist.

Statistics

For all statistical analyses, confirm that the following items are present in the figure legend, table legend, main text, or Methods section.

n/a	Confirmed	
☐	☒	The exact sample size (*n*) for each experimental group/condition, given as a discrete number and unit of measurement
☐	☒	A statement on whether measurements were taken from distinct samples or whether the same sample was measured repeatedly
☐	☒	The statistical test(s) used AND whether they are one- or two-sided<br>*Only common tests should be described solely by name; describe more complex techniques in the Methods section.*
☐	☒	A description of all covariates tested
☐	☒	A description of any assumptions or corrections, such as tests of normality and adjustment for multiple comparisons
☐	☒	A full description of the statistical parameters including central tendency (e.g. means) or other basic estimates (e.g. regression coefficient) AND variation (e.g. standard deviation) or associated estimates of uncertainty (e.g. confidence intervals)
☐	☒	For null hypothesis testing, the test statistic (e.g. *F*, *t*, *r*) with confidence intervals, effect sizes, degrees of freedom and *P* value noted<br>*Give P values as exact values whenever suitable.*
☒	☐	For Bayesian analysis, information on the choice of priors and Markov chain Monte Carlo settings
☒	☐	For hierarchical and complex designs, identification of the appropriate level for tests and full reporting of outcomes
☐	☒	Estimates of effect sizes (e.g. Cohen's *d*, Pearson's *r*), indicating how they were calculated

Our web collection on statistics for biologists contains articles on many of the points above.

Software and code

Policy information about availability of computer code

Data collection	The code is at https://github.com/goodarzilab/SwitchFinder
Data analysis	The code is at https://github.com/goodarzilab/SwitchFinder. The packages used are: UCSF MotionCor2, CTFFIND4, EMAN2 v2.99.47, cryoSPARC v4.4.1, UCSF ChimeraX 1.7.1, cutadapt 4.9, UMI tools 1.1.5, bwa 0.7.18, featureCounts v1.6.2, MPRAnalyze 1.20.0, Pear v0.9.6, CTK 2023.07, DRACO v1.1, HiTRACE, FlowJo v10, FACSDiva v9.0

For manuscripts utilizing custom algorithms or software that are central to the research but not yet described in published literature, software must be made available to editors and reviewers. We strongly encourage code deposition in a community repository (e.g. GitHub). See the Nature Portfolio guidelines for submitting code & software for further information.

Data

Policy information about availability of data

All manuscripts must include a data availability statement. This statement should provide the following information, where applicable:

- Accession codes, unique identifiers, or web links for publicly available datasets
- A description of any restrictions on data availability
- For clinical datasets or third party data, please ensure that the statement adheres to our policy

Sequencing data has been deposited in the Gene Expression Omnibus (GEO accession GSE266070). Cryo-EM density maps have been deposited in EMDB, accession

numbers EMD-42275 (WT Class A), EMD-42276 (WT Class B), EMD-42277 (WT Class C), EMD-42400 (77-GA Class C), EMD-42401 (77-GA Class A), EMD-42403 (117-AC Class C), and EMD-42404 (117-AC Class B). RFAM database 14.10 (https://rfam.org/) was used in the study.

Human research participants

Policy information about studies involving human research participants and Sex and Gender in Research.

Reporting on sex and gender	PBMCs purchased from StemCell are produced from both male and female donors, although specific information on donor gender was not collected for the purpose of this research.
Population characteristics	Peripheral blood mononuclear cells (PBMCs) from anonymous healthy human donors were purchased fresh from StemCell technologies.
Recruitment	PBMCs were purchased from a commercial source: StemCell.
Ethics oversight	PBMCs from anonymous donors were purchased from StemCell Technologies, which collected PBMCs from healthy donors under protocols approved by the StemCell Technologies IRB.

Note that full information on the approval of the study protocol must also be provided in the manuscript.

Field-specific reporting

Please select the one below that is the best fit for your research. If you are not sure, read the appropriate sections before making your selection.

☒ Life sciences ☐ Behavioural & social sciences ☐ Ecological, evolutionary & environmental sciences

For a reference copy of the document with all sections, see nature.com/documents/nr-reporting-summary-flat.pdf

Life sciences study design

All studies must disclose on these points even when the disclosure is negative.

Sample size	For all the high-throughput screens, namely the (i) functional screen, the (ii) structure screen, the (iii) massively parallel mutagenesis screen, and the (iv) CRISPRi screen, the cellular population was maintained at all times with at least 500X coverage of the library size. For CryoEM experiments, the number of particles collected is reported in the Supplementary Tables. For all flow cytometry experiments, data were collected from >10.000 cells to ensure the sufficient coverage.
Data exclusions	No data was excluded from our analyses
Replication	The high-throughput screens, namely the (i) functional screen, the (ii) structure screen, the (iii) massively parallel mutagenesis screen, and the (iv) CRISPRi screen were performed in 2 replicates.<br>DMS-MaP-seq experiments were performed in 2 replicates.<br>SHAPE probing experiments were performed in 3 replicates.<br>CLIP-seq experiments were performed in 2 replicates.<br>Th17 differentiation experiments were performed in 4 replicates.<br>CRISPRi knockdown experiments were performed in 2 replicates.<br>Proteasome inhibition experiments were performed in 3 replicates.<br>qPCR measurements were performed in 2-3 replicates, depending on the experiment.<br>The replicates showed consistent results for all the experiments.
Randomization	The RNA samples were randomly allocated for NMDI14 or DMS treatment, and/or for Bortezomib or Carfilzomib treatment.
Blinding	Where possible, the RT-qPCR step was performed by a different researcher.

Reporting for specific materials, systems and methods

We require information from authors about some types of materials, experimental systems and methods used in many studies. Here, indicate whether each material, system or method listed is relevant to your study. If you are not sure if a list item applies to your research, read the appropriate section before selecting a response.

Materials & experimental systems

n/a	Involved in the study
☐	☒ Antibodies
☐	☒ Eukaryotic cell lines
☒	☐ Palaeontology and archaeology
☒	☐ Animals and other organisms
☒	☐ Clinical data
☒	☐ Dual use research of concern

Methods

n/a	Involved in the study
☒	☐ ChIP-seq
☐	☒ Flow cytometry
☒	☐ MRI-based neuroimaging

Antibodies

Antibodies used

The antibody information is described in the Material and Methods section of the manuscript.
primary
anti-CD4 (Invitrogen 17-0049-42)
anti-FOXP3 (eBioscience 25-4777-61)
anti-IL-13 (eBioscience 11-7136-41)
anti-IL-17A (eBioscience 12-7179-42)
anti-IFNγ (BioLegend 502520)
anti-CD16/CD32 antibody (clone 2.4G2; UCSF Monoclonal Antibody Core AM004)
anti-human CD3 (UCSF monoclonal antibody core, clone: OKT-3, AH003)
anti-human CD28 (UCSF monoclonal antibody core, clone: 9.3, AH002)

Validation

The antibody information is described in the Material and Methods section of the manuscript.
primary
anti-CD4 (Invitrogen 17-0049-42)
Applications Tested: This RPA-T4 antibody has been pre-titrated and tested by flow cytometric analysis of normal human peripheral blood cells. his can be used at 5 µL (0.5 µg) per test. A test is defined as the amount (µg) of antibody that will stain a cell sample in a final volume of 100 µL. Cell number should be determined empirically but can range from 10^5 to 10^8 cells/test.

anti-FOXP3 (eBioscience 25-4777-61)
Applications Tested: This 236A/E7 antibody has been pre-titrated and tested by intracellular staining and flow cytometric analysis of normal human peripheral blood cells using the Foxp3/Transcription Factor Staining Buffer Set (cat. 00-5523) and protocol. Please refer to Best Protocols: Protocol B: One step protocol for (nuclear) intracellular proteins. This can be used at 5 µL (0.125 µg) per test. A test is defined as the amount (µg) of antibody that will stain a cell sample in a final volume of 100 µL. Cell number should be determined empirically but can range from 10^5 to 10^8 cells/test.

anti-IL-13 (eBioscience 11-7136-41)
Applications Tested: This 85BRD antibody has been pre-titrated and tested by intracellular staining followed by flow cytometric analysis of stimulated normal human peripheral blood cells using the Intracellular Fixation & Permeabilization Buffer Set (cat. 88-8824) and protocol. Please refer to Best Protocols: Protocol A: Two step protocol for (cytoplasmic) intracellular proteins located under the Resources Tab online. This can be used at 5 µL (0.25 µg) per test. A test is defined as the amount (µg) of antibody that will stain a cell sample in a final volume of 100 µL. Cell number should be determined empirically but can range from 10^5 to 10^8 cells/test.

anti-IL-17A (eBioscience 12-7179-42)
Applications Tested: This eBio64DEC17 antibody has been pre-titrated and tested by intracellular staining and flow cytometric analysis of stimulated normal human peripheral blood cells. This can be used at 5 µL (0.25 µg) per test. A test is defined as the amount (µg) of antibody that will stain a cell sample in a final volume of 100 µL. Cell number should be determined empirically but can range from 10^5 to 10^8 cells/test.

anti-IFNγ (BioLegend 502520) was tested at https://www.biolegend.com/en-us/products/purified-anti-human-ifn-gamma-antibody-1537?GroupID=BLG2229

All the antibodies purchased from UCSF Monoclonal Antibody Core were validated by UCSF Monoclonal Antibody Core .

Eukaryotic cell lines

Policy information about cell lines and Sex and Gender in Research

Cell line source(s)	HEK293 and Jurkat cells were purchased from ATCC
Authentication	None of the cell lines were authenticated with STR.
Mycoplasma contamination	Cell lines were tested regularly for mycoplasma contamination. No mycoplasma contamination was detected.
Commonly misidentified lines (See ICLAC register)	No commonly misidentified samples were used in this study

Flow Cytometry

Plots

Confirm that:

☒ The axis labels state the marker and fluorochrome used (e.g. CD4-FITC).

☒ The axis scales are clearly visible. Include numbers along axes only for bottom left plot of group (a 'group' is an analysis of identical markers).

☒ All plots are contour plots with outliers or pseudocolor plots.

☒ A numerical value for number of cells or percentage (with statistics) is provided.

Methodology

Sample preparation	For intracellular stains, T cells were fixed and permeabilized with the eBioscience™ Foxp3 / Transcription Factor Staining Buffer Set or the Transcription Factor Buffer Set (BD Biosciences). Extracellular nonspecific binding was blocked with the anti-CD16/CD32 antibody. Intracellular nonspecific binding was blocked with anti-CD16/CD32 Abs and 2% normal rat serum. Finally, up to 0.5 million T cells from culture were washed with PBS + 1% FBS.<br>For HEK293 cells, cells were washed with PBS once, incubated with Trypsin for 10 minutes, then detached from the plate, resuspended in PBS + 1% FBS and strained through industrial mesh with a pore size of 90 uM (ELKO filtering).<br>For Jurkat cells, cells were resuspended in PBS + 1% FBS.<br>For all experiments, known negatives served as gating controls.
Instrument	BD FACSCelesta, BD FACSaria II, BD LSRFortessa
Software	FlowJo 10.7.1 and BD FACSDiva v9
Cell population abundance	All sorts were end-point sorts and not for subsequent culture.
Gating strategy	For all flow cytometry data, viable cells were gated by FSC-A/SSC-A (as well as live/dead cell markers for some experiments), and singlets by FSC-A/FSC-H. Positive populations were determined by unstained (in case of T cells) or non-transduced (in case of Jurkat or HEK293) samples. Gating strategy is shown in the supplementary data.

☒ Tick this box to confirm that a figure exemplifying the gating strategy is provided in the Supplementary Information.
